# Matrix Metalloproteinase 9 Gene Promoter (rs 3918242) Mutation Reduces the Risk of Diabetic Microvascular Complications

**DOI:** 10.3390/ijerph120708023

**Published:** 2015-07-14

**Authors:** Zhongwen Zhang, Xiaoyun Wu, Tian Cai, Weiyi Gao, Xiaojun Zhou, Junyu Zhao, Jinming Yao, Hongxia Shang, Jianjun Dong, Lin Liao

**Affiliations:** 1 Department of Medicine, Shandong Provincial Qianfoshan Hospital, Shandong University, 16766 Jingshi Road, Jinan 250014, China; E-Mail: zhangzhongwen521@126.com; 2 Division of Endocrinology, Department of Medicine, Shandong Provincial Qianfoshan Hospital, Shandong University, Jinan 250014, China; E-Mails: jkeliya@163.com (X.W.); caitianzhi900205@163.com (T.C.); 1989919zm@163.com (X.Z.); junyuzhao07@gmail.com (J.Z.); jinmingvvip@sina.com (J.Y.); shanghongxia1989@foxmail.com (H.S.); 3 Department of Cadres Healthcare, Qilu Hospital of Shandong University, Qingdao 266035, China; E-Mail: gaoweiyiqdql@sina.com; 4 Division of Endocrinology, Department of Medicine, Qilu Hospital of Shandong University, Jinan 250012, China

**Keywords:** matrix metalloproteinase 9, diabetes mellitus, meta-analysis

## Abstract

*Background:* Many studies have evaluated the association between matrix metalloproteinase 9 (MMP9) gene promoter polymorphism and diabetic microvascular complications. However, the results are conflicting and inconclusive. The aim of this meta-analysis was to evaluate the association more precisely. *Materials and Methods:* Studies were retrieved from the PubMed, Embase, Medline, China National Knowledge Infrastructure, Web of Science, and Cochrane databases. All statistical analyses were performed using Review Manager 5.2. *Results:* Data were abstracted from four case-control studies that included 446 patients with diabetic microvascular complications and 496 diabetic control subjects. The MMP9-1562 C/T genotype was significantly associated with the risk of diabetic nephropathy after stratification by specific type of microvascular complication (CT + TT *vs.* CC: OR = 0.42, 95% CI = 0.26–0.69, *p* = 0.0006; TT *vs.* CC + CT: OR = 0.37, 95% CI = 0.19–0.76, *p* = 0.006). *Conclusions:* This study adds to the evidence that MMP9-1562 T gene mutation might reduce the risk of diabetic nephropathy.

## 1. Introduction

Diabetic microvascular complications (DMI), mainly including diabetic retinopathy (DR) and diabetic nephropathy (DN), have been increasing steadily around the world [1,2,3,4,5]. Although the precise mechanisms for the pathogenesis of DMI are still undefined, many risk factors, such as the duration of diabetes and the degrees of glycemic and blood pressure control, are identified in the causation of DMI [6,7]. Additionally, several gene polymorphisms, such as superoxide dismutase (SOD), endothelial nitric oxide synthase (eNOS), suprachiasmatic nucleus 9A (SCN9A), vascular cell adhesion molecule-1(VCAM-1), vascular endothelial growth factor (VEGF), olfactory receptor 4K17 (OR4K17), apolipoprotein C1 (APOC1), basic fibroblast growth factor (bFGF), insulin-like growth factor (IGF), and matrix metalloproteinases (MMPs) [8,9,10,11,12,13,14,15,16,17,18] have been indicated as contributing factors in prior studies. Therefore, finding a genetic marker for DMI would be important in identifying patients who could benefit from preventive treatment.

Hyperglycemia-induced changes in microvascular wall structure contribute to the pathological process of DMI, which is characterized by thickening of the capillary basement membranes and increased deposition of extracellular matrix components (ECM), while loss of microvessels with subsequent neovascularization is predominant in the eye, kidney, and peripheral nerves [19]. Matrix metalloproteinase 9 (MMP9), a member of the gelatinase family of MMPs that degrade ECM proteins, plays an important role in microvascular remodeling. MMP9 expression is up-regulated in the lesions of the vascular wall during the process of DMI, and its high expression is associated with the recruitment of leukocytes to lesions and fibrosis of the microvascular wall [20,21]. Variants in the regulatory regions of *MMP9* gene have been considered to be an important factor that influences *MMP9* expression [22,23]. A number of epidemiological studies have evaluated the association between the genetic variants of the *MMP9* gene and the risk of DMI [24,25,26]. The most common variant is MMP9-1562C/T promoter polymorphism (rs 3918242) [9,10,11]. However, the results were controversial. Some researchers have indicated that the functional -1562 C-to-T polymorphism in *MMP9* gene promoter was associated with the risk of DMI [24,27,28]. Yet, the results have not been verified in other studies [26]. Since a single-case study may lack of the power to provide dependable conclusions, we carried out this meta-analysis to evaluate the exact effect of the *MMP9*-1562C/T gene promoter variant on the risk of DMI.

## 2. Experimental Section

### 2.1. Search Strategy

We searched the following electronic databases: PubMed, Embase, Web of Science, China National Knowledge Infrastructure, Cochrane database, and the references list of relevant studies. We used these search terms: “Matrix metalloproteinase 9”, “MMP9”, or “rs3918242” in combination with “diabetes mellitus” or “diabetic microvascular complications” or "diabetic nephropathy" or “diabetes kidney disease” or “diabetic retinopathy” or “ diabetic dermopathy ” or “variant” or “mutation” or “single nucleotide polymorphism” or “SNP” or “polymorphism”. There was no restriction on language or whether or not the articles had been published.

### 2.2. Inclusion Criteria and Information Extracted

Eligible studies met the following criteria: (1) Case-control study, patients with diabetic microvascular complications as case group, diabetic patients without complications as control group; (2) Studies evaluating the association between the MMP9-1562C/T promoter polymorphism and the risk of DMI; (3) Studies with sufficient data for calculating odds ratios (ORs) and 95% confidence intervals (95% CIs). Exclusion criteria included: (1) Kidney damage due to diseases other than diabetes; (2) Animal studies.

### 2.3. Data Extraction

Two authors (ZWZ and JJD) independently screened abstracts and extracted data from included studies according to the standard technological procedure [8,29]. Table 1 lists the characteristics of the extracted data.

**Table 1:** Main characteristics of the studies included in the meta-analysis.

First author	Ethnicity	Subjects, N cases/controls	Mean age, y cases/controls	Genotypes (CC/CT/TT)	% of male (cases/controls)	Test for HWE X^2^ *P*
				cases/controls		
**ML Pang *et al.* (2010)**	Asian	150/52	57.4 ± 14.1/ 58.7 ± 15.3	77/30/43 24/6/22	52.5/22.4	29.3 *p* < 0.05
**M Beránek *et al.* (2008)**	Caucasian	304/186	62.3 ± 0.7/ 59.4 ± 0.9	220/79/5 134/49/3	NA/NA	1.08 *p* *=* 0.29
**K Singh *et al.* (2013)**	Caucasian	259/267	55.3 ± 8.9/ 51.4 ± 10.5	164/91/4 196/69/2	85.0/85.2	2.56 *p* *=* 0.10
**KZ Liu *et al.* (2003)**	Asian	230/110	63.4 ± 9.2/ 64.2 ± 11.0	196/31/3 85/23/2	57.4/45.5	0.16 *p* *=* 0.68

N, Number of studies; NA, Not available; HWE: Hardy-Weinberg equilibrium.

### 2.4. Statistical Analysis

The strength of the association between *MMP9* gene mutation and DMI was evaluated by ORs and their corresponding 95% CIs. If the association exhibited high heterogeneity (I^2^ > 25%), the random effects model was merged. Otherwise, the fixed effects model was used. The HWE was tested by the X^2^ test. The potential publication bias was estimated using funnel plots and Rosenthal's fail-safe number (Nfs). Rosenthal's fail safe number is calculated via the formula Nfs0.05 = (ΣZ/1.64)^2^ – k (k is the number of articles included in this study). The association between the MMP9-1562 C/T gene mutation and DMI was analyzed in the dominant model (T positive genotype) by CT + TT *vs.* CC; in the recessive model (T negative genotype), TT *vs.* CT + CC was used. The risk frequency of the T allele was calculated in these case-control groups. The ethnicity (Caucasians or Asians) and the specific type of microvascular complication (diabetic nephropathy, diabetic retinopathy or diabetic dermopathy) were selected for stratified analysis. All analyses were performed using Review Manager 5.2 (Cochrane Collaboration, Oxford, UK). In subgroup analyses *p* values were Bonferroni adjusted to account for multiple testing. If the corrected *p*-value is still below the error rate, the gene will be significant: Corrected *p*-value = *p*-value * n (number of groups in test) < 0.05.

## 3. Results and Discussion

### 3.1. Study Characteristics

**Figure 1:**
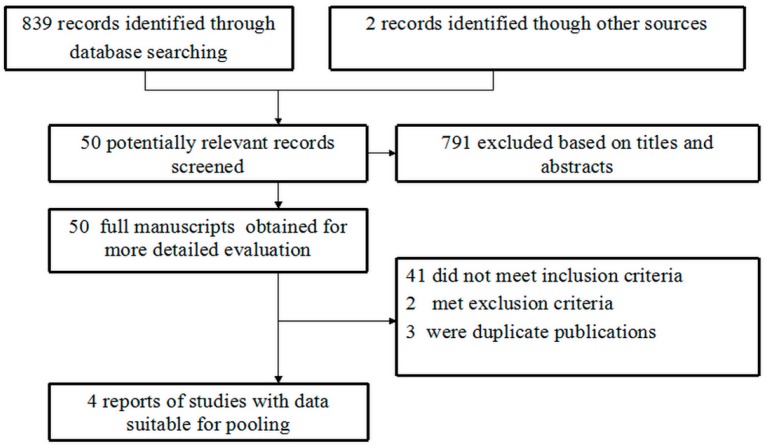
Flow diagram of paper selection for the meta-analysis.

The study selection process is indicated in Figure 1. In brief, 839 records were identified by database searches and 2 through other sources, of which 791 were excluded after scanning the titles and abstracts.

Two investigators (Z.Z. and J.D.) reviewed the full articles of the remaining 50 abstracts and excluded 46 articles. Four original studies satisfied the selection criteria and were included in our meta-analysis. The main characteristics of the included studies are listed in Table 1. All the studies involved a hospital-based design. Two studies [24,26] investigated Caucasian population and two studied [27,28] Asians. The percentages of MMP9-1562 T allele carriers ranged from 4.6 % to 30.1% in DMI patients and 12.5% to 47.9 % in controls. Except for the Pang *et al.* study [28] (*p* < 0.05 for controls), the genotype distributions for both patients and controls were in HWE (*p* > 0.05).

### 3.2. Main Meta-Analysis Results

**Figure 2:**
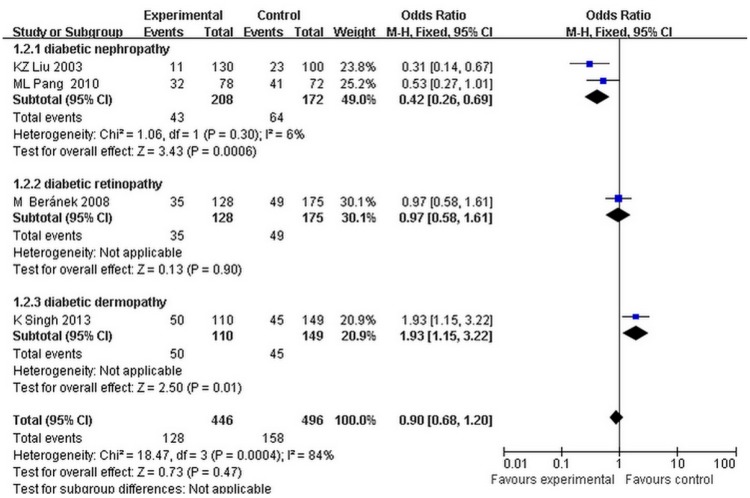
Forest plots of the meta-analysis for MMP9-1562C to T gene mutation (CT + TT *vs.* CC) associated with the subtypes of diabetic microvascular complications.

The fixed effects model was used because the heterogeneity of the data was less than 25%. It showed that the MMP9-1562 C/T genotype was significantly associated with the risk of diabetic nephropathy, after stratification by specific type of microvascular complication (dominant model: OR = 0.42, 95% CI = 0.26–0.69, *p* = 0.0006, Corrected *p*-value = 0.0018, heterogeneity: I^2^ = 6%; recessive model: OR = 0.37, 95% CI = 0.19–0.76, *p* = 0.006, Corrected *p*-value = 0.018, heterogeneity: I^2^ = 0%) (Figure 2).

There was a significant association between MMP9-1562 C/T genotype and DMI in Asians (dominant model: OR = 0.42, 95% CI = 0.26–0.69, *p* = 0.0006, corrected *p*-value = 0.0012, heterogeneity: I^2^ = 6% for Asians; OR = 1.36, 95% CI = 0.95–1.95, *p* = 0.09, corrected *p*-value = 0.18, heterogeneity: I^2^ = 71% for Caucasians; recessive model: OR = 0.37; 95% CI = 0.19–0.76, *p* = 0.006, Corrected *p*-value = 0.018, heterogeneity: I^2^ = 0% for Asians; OR = 2.77, 95% CI = 0.69–11.18, *p* = 0.15, Corrected *p*-value = 0.30, heterogeneity: I^2^ = 0% for Caucasians). In addition, we calculated the ‘T’ allelic frequency of MMP9 -1562 C/T genotype in Asians and Caucasians. In the Caucasian population, the frequency of the ‘T’ allele in the case group was lower than that in control group (case group: mean = 0.150038, range = 0.145714–0.154362; control group: mean = 0.194098, range = 0.147287–0.240909). However, in the Asian population, the frequency of the ‘T’ allele in the case group was higher than that in control group (case group: mean = 0.3020835, range = 0.125–0.479167; control group: mean = 0.173718, range = 0.046154–0.301282).

### 3.3. Publication Bias

**Figure 3:**
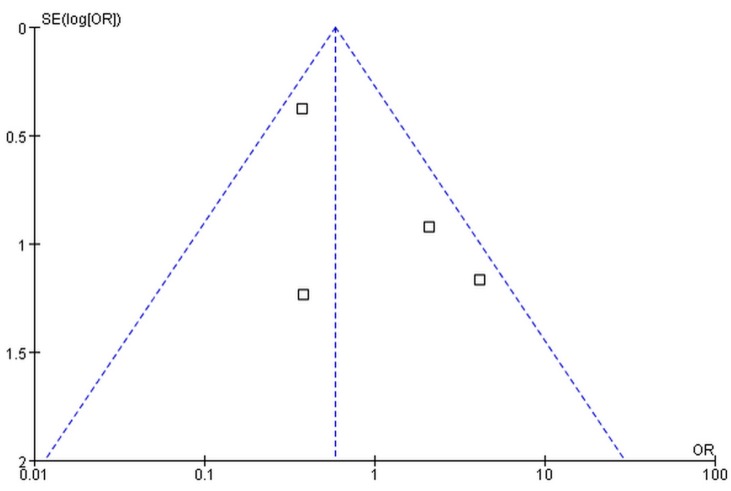
Funnel plot of publication bias for the association of MMP9 -1562C/T gene polymorphism with the risk of diabetic microvascular complications.

The publication bias was assessed by funnel plot and Nfs0.05. No significant publication bias was found (Figure 3). The Nfs0.05 value of each comparison was greater than the number of studies included in our meta-analysis.

### 3.4. Sensitivity Analysis

**Figure 4:**
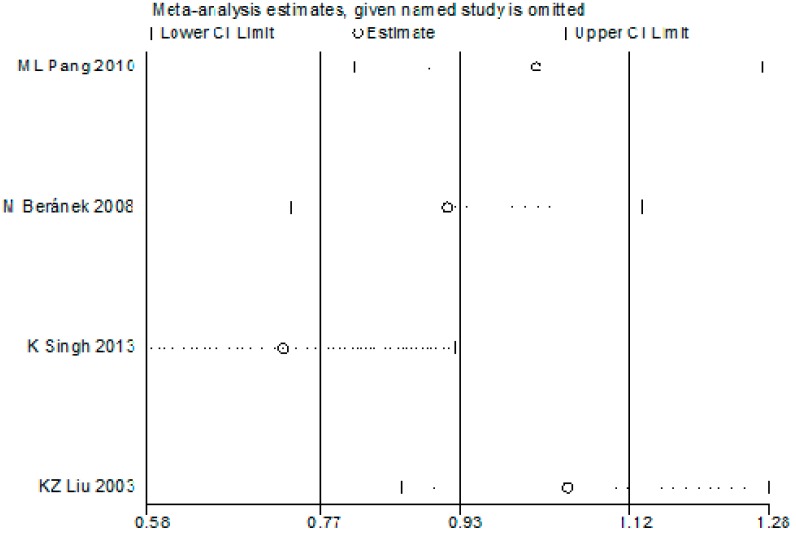
The sensitivity analysis for the association of MMP9-1562C/T gene polymorphism with risk of diabetic microvascular complications.

In consideration of the fact that the controls in the study by Pang *et al*. were not in HWE [28], sensitivity analysis was performed to assess the impact of this study on the overall conclusions. As shown in Figure 4, the genotype distribution of the control groups of the Pang *et al*. study deviated significantly (*p* < 0.05) from HWE. The meta-analysis based on corrected estimates for the random effects OR (OR _adjusted_) for the dominant model for allele T produces an estimate of OR _adjusted_ = 0.93 (0.77–1.12), whereas the raw overall analysis and the sensitivity analysis produce similar estimates: OR = 1.01 (0.81–1.26). The result indicated that the departure from HWE in Pang’s study has no major impact.

### 3.5. Discussion

To the best of our knowledge, this is first meta-analysis examining the relationship between MMP9-1562 C/T gene mutation and DMI susceptibility. Our subtype analyses found a significant association between the MMP9-1562 T gene mutation and the risk of diabetic nephropathy in Asians.

Previous studies have suggested that microvascular remodeling might affect the development of diabetic nephropathy by degradation products of extracellular matrix proteins in the mesangium and basement membrane of the glomerulus [30]. MMP9, a major regulator of the extracellular matrix, is capable of degrading the extracellular matrix proteins. In a previous preliminary study, Uemura *et al*. [31] reported a significant correlation between MMP9 and diabetic vascular disease. Liu *et al*. [27] suggested MMP9 as an indicator of renal microvascular remodeling in patients with diabetic nephropathy. Polymorphisms in the promoter of the MMP9 gene have been shown to significantly affect the transcriptional activity and the release of MMP9. For example, the -1562C/T polymorphism is associated with an increased transcriptional activity and high expression of MMP9. Thus, the -1562C/T polymorphism of MMP9 gene may represent a candidate to identify genetic risk factors for diabetic nephropathy. In this meta-analysis, a total of four studies, including 446 DMI cases and 496 diabetic controls, were collected to evaluate the association between MMP9-1562C/T gene promoter polymorphism and the risk of DMI. Disappointedly, there was no significant association between MMP9-1562C/T gene promoter polymorphism and the risk of DMI under dominant and recessive models. However, in the subtype analysis by the specific type of microvascular complication, a significant association was found between MMP9-1562C/T gene polymorphism and the risk of diabetic nephropathy under dominant and recessive models. Since only a few of these studies are available, these conclusions have to be confirmed further by large population studies.

Some limitations should be considered in this meta-analysis. First, the number of subjects is relatively small, and additional case-control studies are needed to confirm the conclusion. Second, the DN study populations were Asians, and study populations from other ethnic groups did not exist. Therefore, more studies should be conducted in other ethnic groups to increase the statistical power. Third, the MMP9 gene is just one of a host of genetic risk factors for DN; other MMP genes, such as MMP2, MMP8, and MMP10 may also participate in the pathogenesis of DN. Moreover, considering that positive results are much more likely to be published, negative findings in research may be suppressed [32]. Although no evidence of publication bias was found, the number of meta-analysis studies was small and inter-study heterogeneity was observed. The heterogeneity could be due to differences in several factors such as the duration of diabetes, subtype of diabetes mellitus and the type of microvascular complication. In addition, DMI involves combined effects of multiple genetic and environmental factors and different ethnic populations have different genetic backgrounds, which may affect DMI susceptibilities [8,33]. In our study, after excluding the trials of Beránek [26] and Singh [24], the heterogeneity was reduced (dominant models: I^2^ = 6%, recessive models: I^2^ = 0%) and a significant association was found between MMP9-1562C/T gene polymorphism and the risk of DN. In addition, the heterogeneity was reduced after excluding the Caucasian population. These results indicated that the type of microvascular complication and ethnicity were correlated with heterogeneity. However, all of the Asian studies involved diabetic nephropathy instead of diabetic retinopathy or diabetic dermopathy. It is unclear whether the existence of heterogeneity is due to the differences in the type of microvascular complications or ethnicity and additional studies should be performed in the future to dismiss the confounding factors before arriving at conclusions.

## 4. Conclusions

In summary, our study provided potential evidence of the association between MMP9-1562C/T gene promoter polymorphism and the risk of DMI, and supported the hypothesis that the MMP9-1562 T allele variant might be a genetic marker for the risk of DN. Since the sample size of MMP9 polymorphism studies is relatively small, additional case-control studies with large sample sizes are required to validate our findings. In addition, further studies concerning other single nucleotide polymorphisms in the MMP9 gene and DMI susceptibility are also encouraged to clarify the role of the MMP9 gene in DMI.
